# Toll‐like receptor 4 regulates spontaneous intestinal tumorigenesis by up‐regulating IL‐6 and GM‐CSF

**DOI:** 10.1111/jcmm.14742

**Published:** 2019-10-25

**Authors:** Yun‐Jie Shi, Quan‐Quan Zhao, Xiao‐Shuang Liu, Su‐He Dong, Ji‐Fu E, Xu Li, Cong Liu, Hao Wang

**Affiliations:** ^1^ Department of Colorectal Surgery Chang Hai Hospital Second Military Medical University Shanghai China; ^2^ Department of Radiation Medicine Faculty of Naval Medicine Second Military Medical University Shanghai China

**Keywords:** adenomatous polyposis coli, GM‐CSF, IL‐6, spontaneous intestinal tumorigenesis, Toll‐like receptor 4

## Abstract

Inflammation is as an important component of intestinal tumorigenesis. The activation of Toll‐like receptor 4 (TLR4) signalling promotes inflammation in colitis of mice, but the role of TLR4 in intestinal tumorigenesis is not yet clear. About 80%–90% of colorectal tumours contain inactivating mutations in the adenomatous polyposis coli (Apc) tumour suppressor, and intestinal adenoma carcinogenesis in familial adenomatous polyposis (FAP) is also closely related to the germline mutations in Apc. The Apc^Min/+^ (multiple intestinal neoplasia) model mouse is a well‐utilized model of FAP, an inherited form of intestinal cancer. In this study, Apc^Min/+^ intestinal adenoma mice were generated on TLR4‐sufficient and TLR4‐deficient backgrounds to investigate the carcinogenic effect of TLR4 in mouse gut by comparing mice survival, peripheral blood cells, bone marrow haematopoietic precursor cells and numbers of polyps in the guts of Apc^Min/+^ WT and Apc^Min/+^ TLR4^−/−^ mice. The results revealed that TLR4 had a critical role in promoting spontaneous intestinal tumorigenesis. Significant differential genes were screened out by the high‐throughput RNA‐Seq method. After combining these results with KEGG enrichment data, it was determined that TLR4 might promote intestinal tumorigenesis by activating cytokine‐cytokine receptor interaction and pathways in cancer signalling pathways. After a series of validation experiments for the concerned genes, it was found that IL6, GM‐CSF (CSF2), IL11, CCL3, S100A8 and S100A9 were significantly decreased in gut tumours of Apc^Min/+^ TLR4^−/−^ mice compared with Apc^Min/+^ WT mice. In the functional study of core down‐regulation factors, it was found that IL6, GM‐CSF, IL11, CCL3 and S100A8/9 increased the viability of colon cancer cell lines and decreased the apoptosis rate of colon cancer cells with irradiation and chemical treatment.

## 1 INTRODUCTION

Colorectal cancer (CRC) has become the second leading cause of cancer‐related death in developed countries. The transitional treatments include surgery, combined chemotherapy and radiation therapy.^1^ Currently, the use of targeted therapies is limited to patients with stage IV metastatic colon cancer.^2^ Thus, the identification of new targets for CRC therapy is needed.

Previous studies have reported that the Apc/Wnt pathway is an attractive target for the treatment of CRC.^3^ The adenomatous polyposis coli (Apc) tumour suppressor is mutated in 80%–90% of human colorectal tumours.^4^ Apc, along with axin and glycogen synthase kinase 3b (GSK3b), assembles a protein complex that targets β‐catenin for degradation, which is regulated by Wnt signalling.^5^ This is the main mechanism of Apc mutation leading to tumorigenesis, as the resulting increased availability of β‐catenin causes changes in the transcriptional process to decrease the amount of cell differentiation.^6^

TLRs are sensors for pathogen‐associated molecular patterns (PAMPs) and play important roles in initializing inflammation.^7^ Toll‐like receptor 4 (TLR4) is the main mediator of responses to lipopolysaccharide (LPS) both in vitro and in vivo.^8^ It has been reported that inflammatory responses contribute to intestinal carcinogenesis through multiple mechanisms, for example TLR4 signalling activates NF‐κB through the MYD88 pathway,^9^, ^10^, ^11^ and it is well known that patients with inflammatory bowel disease are at higher risk of CRC.^12^TLR4/MYD88 signalling contributes to CRC tumorigenesis not only in colitis‐associated cancer, but also in sporadic CRC.^13^ Disorders of intestinal microflora can also lead to intestinal carcinogenesis. https://www.ncbi.nlm.nih.gov/pubmed/?term=Chen%2520Y%255BAuthor%255D%26cauthor=true%26cauthor_uxml:id=28423670 et al reported that *Fusobacterium nucleatum *activates beta‐catenin signalling in CRC via a TLR4/P‐PAK1 cascade.^14^ However, it has been reported that TLR4‐deficient C3H/HeJ mice developed more tumours relative to TLR4‐normal C3H/HeN mice.^15^ This suggests that TLR4 may also have a role in inhibiting tumorigenesis. It is therefore evident that the specific regulation mechanism of TLR4 signalling that promotes or inhibits tumorigenesis is unclear. In the present study, Apc^Min/+^ (multiple intestinal neoplasia) mice were generated on TLR4‐sufficient and TLR4‐deficient backgrounds to explore the role of this pathway in spontaneous intestinal tumorigenesis. Apc^Min/+^ mice carry a single mutant Apc allele and develop 10‐55 benign adenomas in the gut by 4‐5 months of age.^3^ In this study, high‐throughput sequencing was used to screen out the production of TLR4 signalling downstream genes and validate them in a variety of ways. These data may provide an insight into the role of TLR4 in intestinal tumorigenesis.

## 2 METHODS

### 2.1 Mice

Adult WT mice (C57BL/6 wild type) were purchased from the Chinese Academy of Science (Shanghai, China). TLR4^−/−^, Apc^Min/+^ WT and Apc^Min/+^ TLR4^−/−^ mice were provided by the Model Animal Research Center of Nanjing University (Nanjing, China).

All mice were bred and maintained under specific pathogen‐free (SPF) conditions in the laboratory of the Animal Center of Second Military Medical University. In the survival studies, the number of mice per group is shown in each figure, and 3 mice per group were used in all other experiments. RNA‐Seq analysis included 3 pairs of colon samples of Apc^Min/+^ WT and Apc^Min/+^ TLR4^−/−^ mice. All the mice used in the experiments were 4‐6 months old. This study was approved by an ethics committee and followed the tenants of the Declaration of Helsinki.

### 2.2 Cell culture

CT‐26 cells^16^ (mouse intestinal cancer cell lines) and SW1116 cells 17 (human intestinal cancer cell lines) used in this study were obtained from the Shanghai Institute of Biochemistry and Cell Biology, Chinese Academy of Sciences (Shanghai, China). Cells were cultured in Dulbecco's modified Eagle's medium (DMEM; PAA, Austria) containing 10% foetal calf serum (PAA) at 37°C in a humidified atmosphere of 5% CO_2_. All cell lines used in this study had been cultured for less than 6 months when the experiments were performed.

### 2.3 Analysis of peripheral blood

To explore the peripheral blood conditions, the mice were eyebled using heparin‐coated capillary tubes to take blood while the mice were under anaesthesia. The blood was transferred to Eppendorf (EP) tubes with K2‐EDTA and inverted multiple times. A small animal blood cell counter was used to measure blood cells.

### 2.4 Measurement of bone marrow haematopoietic precursor cells

Femur bone marrow was taken from each group of mice to make bone marrow nucleated cell suspensions. After the bone marrow nucleated cell suspensions were diluted to the appropriate concentration, the corresponding fluorescently labelled antibody was added in a ratio of 1:100 (GMP: Lin^−^c‐Kit^+^FcγRII/III^hi^CD34^hi^; CMP: Lin^−^c‐Kit^+^FcγRII/III^lo^CD34^hi^; MEP: Lin^−^c‐Kit^+^FcγRII/III^lo^CD34^lo^; EP1: Ter119^−^CD71^hi^; EP2: Ter119^hi^CD71^hi^; EP3: Ter119^hi^CD71^med^; EP4: Ter119^hi^CD71^lo^). A CD34‐FcγRII/III kit and Ter119‐CD71 kit were provided by the eBioscience Corporation (USA). The suspensions were mixed well and incubated for 20 min at room temperature in the dark; PBS was then added to mix and dilute, and flow cytometry (Beckman Coulter, USA) was used for testing.^18^, ^19^

### 2.5 Histological study

Gut tissues were dehydrated in an ascending grade of ethanol, then cleared and embedded in paraffin wax. Serial sections of 2‐7 microns thick were obtained using a rotatory microtome. The deparaffinized sections were stained routinely using the H & E staining method. Ki67 kits were applied to assess the proliferative capacity of intestinal epithelial crypt cells.^20^ TUNEL kits were applied to assess the apoptosis of intestinal epithelial crypt cells.^21^ Photomicrographs of the desired sections were obtained using a digital research photographic microscope (Thermo Corporation, USA).

### 2.6 RNA‐Seq analysis

The project was undertaken by Oebiotech Corporation (Shanghai, China). Total RNA was extracted using a mirVana miRNA Isolation Kit. RNA integrity was evaluated using an Agilent 2100 Bioanalyzer (Agilent Technologies, Santa Clara, CA, USA). The samples with an RNA integrity number ≥7 were subjected to subsequent analysis. The libraries were constructed using a TruSeq Stranded mRNA LTSample Prep Kit (Illumina, San Diego, CA, USA). These libraries were then sequenced on the Illumina sequencing platform (HiSeqTM 2500 or Illumina HiSeq X Ten), and 125/150 bp paired‐end reads were generated. A *P*‐value <.05 and fold change >2 or <.5 was set as the threshold for significant differential expression. Hierarchical cluster analysis of differentially expressed transcripts (DEGs) was performed to explore gene expression patterns. KEGG 22 pathway enrichment analyses of DEGs were, respectively, performed using *R* based on the hypergeometric distribution. After the differentially expressed genes were obtained, the pathways with numbers of differential genes greater than 2 were screened, and KEGG enrichment analysis was performed to screen out the top 20 total pathways with the values of the ‐log10 *P*‐value of each pathway. The accession numbers of the sequence read archive (SRA) database for the RNA‐Seq data were as follows: sampleApc1 SRR10001234, sampleApc2 SRR10001235, sampleApc3 SRR10001232, sampleTLR4_Apc1 SRR10001238, sampleTLR4_Apc2 SRR10001239, sampleTLR4_Apc3 SRR10001236.

### 2.7 Quantitative real‐time PCR (RT‐qPCR)

Total RNAs from tissue samples were isolated using an RNA isolation and purification reagent kit provided by GenePharma Corporation (Shanghai, China). All the primers were synthesized by Sangon Biotech Corporation (Shanghai, China). cDNA was synthesized using a Hifair^TM^II 1st‐Strand cDNA Synthesis SuperMix RT reagent kit (YeaSen Biotech, Shanghai, China). Fluorescence quantitative real‐time PCR reactions were performed using a SYBR Premix EX Taq Kit (TaKaRa, Dalian, China).^23^

### 2.8 Cytokine measurement via enzyme‐linked immunosorbent assay (ELISA)

The amounts of cytokines in the serum of mice or CT‐26 cells were measured by ELISA kits provided by Dakewe Biotech Corporation (Shenzhen, China). To determine the factors produced by polyps in the gut, each colon with tumours was divided into three segments of approximately 1 cm each and washed in phosphate‐buffered saline (PBS) supplemented with penicillin and streptomycin (Gibco from Hyclone Corporation, USA). These segments were cultured in 24‐well flat‐bottom culture plates in serum‐free 1640 medium (Gibco) supplemented with penicillin and streptomycin. After 24 hours, the supernatant fluid was collected and stored at 20°C. Cytokines in the supernatant were measured via ELISA kits provided by Dakewe Biotech Corporation (Shenzhen, China) and were normalized for the number of cytokines per mg of total protein in the supernatant.^24^ In the experiments of IL‐6 and GM‐CSF mediated by TLR4, the mice were abdominally injected with 1 mg/kg bodyweight LPS, and the CT‐26 cells were treated with 1 ng/mL of LPS 24 hours before measuring the IL6 and GM‐CSF with the ELISA kit.

### 2.9 Flow multifactor detection assay

The mean fluorescence intensity of the cytokines in the serum of each group was measured by a multifactor detection reagent kit 25 (Dakewe Biotech Corporation, Shenzhen, China), and flow cytometry (Beckman Coulter, USA) was utilized for testing.

### 2.10 MTT assay

Relative cell viability was analysed using tetrazolium salt 3–(4, 5‐dimethylthiazol‐2‐yl)22, 5‐diphenyltetrazolium bromide(MTT) (Dojindo Corporation, Shanghai, China). The CT‐26 cells were treated with 100 ng/mL of LPS, IL6, GM‐CSF, IL11, CD40, TNF, CCL3, MMP9 and S100A8/A9 24 hours before MTT analysis. The SW1116 cells were treated with 100 ng/mL of LPS, IL6, GM‐CSF and IL11 24 hours before MTT analysis. For analysis, 20 μl of MTT substrate was added to each well. The plates were returned to the incubator for an additional 4 hours at 37°C with 5% CO_2_. The medium was removed, and the cells were solubilized in 150 μl of dimethyl sulphoxide before colorimetric analysis was performed (wavelength: 490 nm). One plate was analysed immediately after the cells adhered (approximately 4 hours after plating), and the remaining plates were analysed every day for the next 3 days.^26^

### 2.11 Apoptosis analysis

Cell apoptosis was analysed using apoptosis detection kits^27^ (TransGen Biotech, Beijing, China). The CT‐26 cells were treated with 100 ng/mL of LPS, IL6, GM‐CSF, IL11, CD40, TNF, CCL3, MMP9 and S100A8/A9 for 24 hours, and were then exposed to 6 Gy radiation irradiation or treatment with the chemotherapy drug paclitaxel. The SW1116 cells were treated with 100 ng/mL of LPS, IL6, GM‐CSF and IL11 for 24 hours, and were then exposed to 6 Gy radiation irradiation or treatment with the chemotherapy drug paclitaxel. After 24 hours, the CT‐26 and SW1116 cells were stained with Annexin V‐FITC/propidium (PI) (eBioscience Corporation, USA), and cell apoptosis rates were detected by flow cytometry (Beckman Coulter, USA). γ radiation at a rate of 1.8 Gy/min in a ^60^Co irradiator was provided by the Department of Radiation Medicine of Second Military Medical University. The treatment concentration of paclitaxel (MedChemExpress, USA) was 0.05 μmol.

### 2.12 Amounts of MDSC analysis in spleen and bone marrow of mice

Femur bone marrow was taken from each group of mice to make bone marrow nucleated cell suspensions (BMCs). The spleen taken from each group of mice was mashed. After the BMCs and spleen suspensions were diluted to the appropriate concentration, the corresponding fluorescently labelled antibody CD11b/Gr_1_ (eBioscience Corporation, USA) was added. The suspensions were mixed well and incubated for 20 min at room temperature in the dark; PBS was then added to mix and dilute, and flow cytometry (Beckman Coulter, USA) was used for testing.

### 2.13 Statistical analysis

Comparisons among experimental groups and relevant controls (but not survival studies) were performed using Student's t test or one‐way analysis of variance. Survival studies were performed on GraphPad Prism software (GraphPad Software Inc, La Jolla, CA, USA). Differences in survival studies of the various groups of mice were assessed using the Kaplan‐Meier method and Cox regression analysis. Statistical analysis generated a *P*‐value and statistic value for each analysis; *P* < .05 was considered a statistically significant difference.

## 3 RESULTS

### 3.1 TLR4^−/−^ inhibited spontaneous intestinal tumorigenesis in Apc^Min/+^ model mice

**Figure 1:**
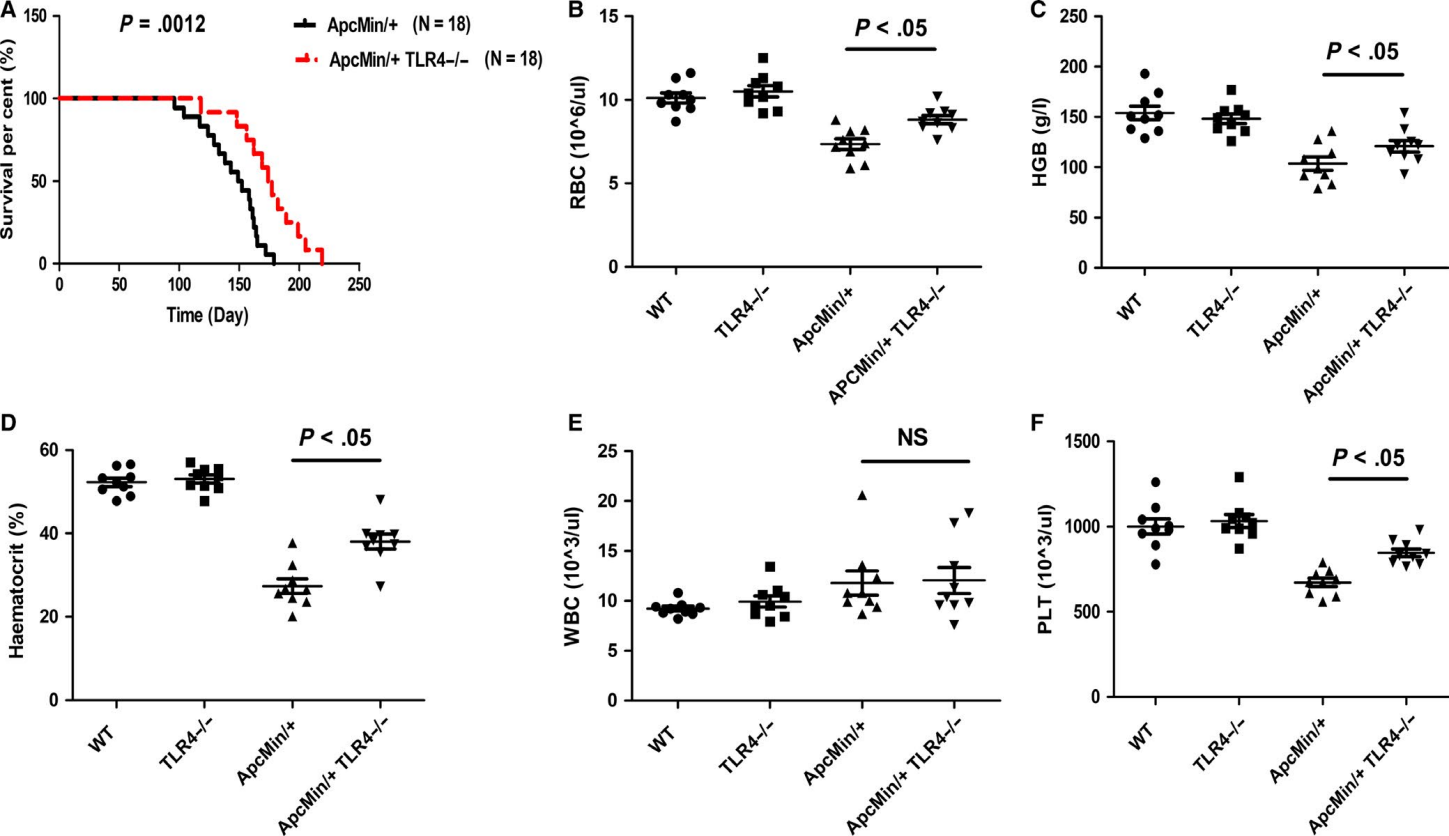
Effects of TLR4 deficiency in ApcMin^/+^ mice on mortality and peripheral blood cells. (A) Apc^Min/+^ WT mice (N = 18) and Apc^Min/+^ TLR4^−/−^ mice (N = 18) were passively followed for long‐term survival. The survival was recorded. (B‐F) At 20‐22 weeks of age, the peripheral blood cells of each group of mice were determined. *P < .05; NS: No significant difference detected

**Figure 2:**
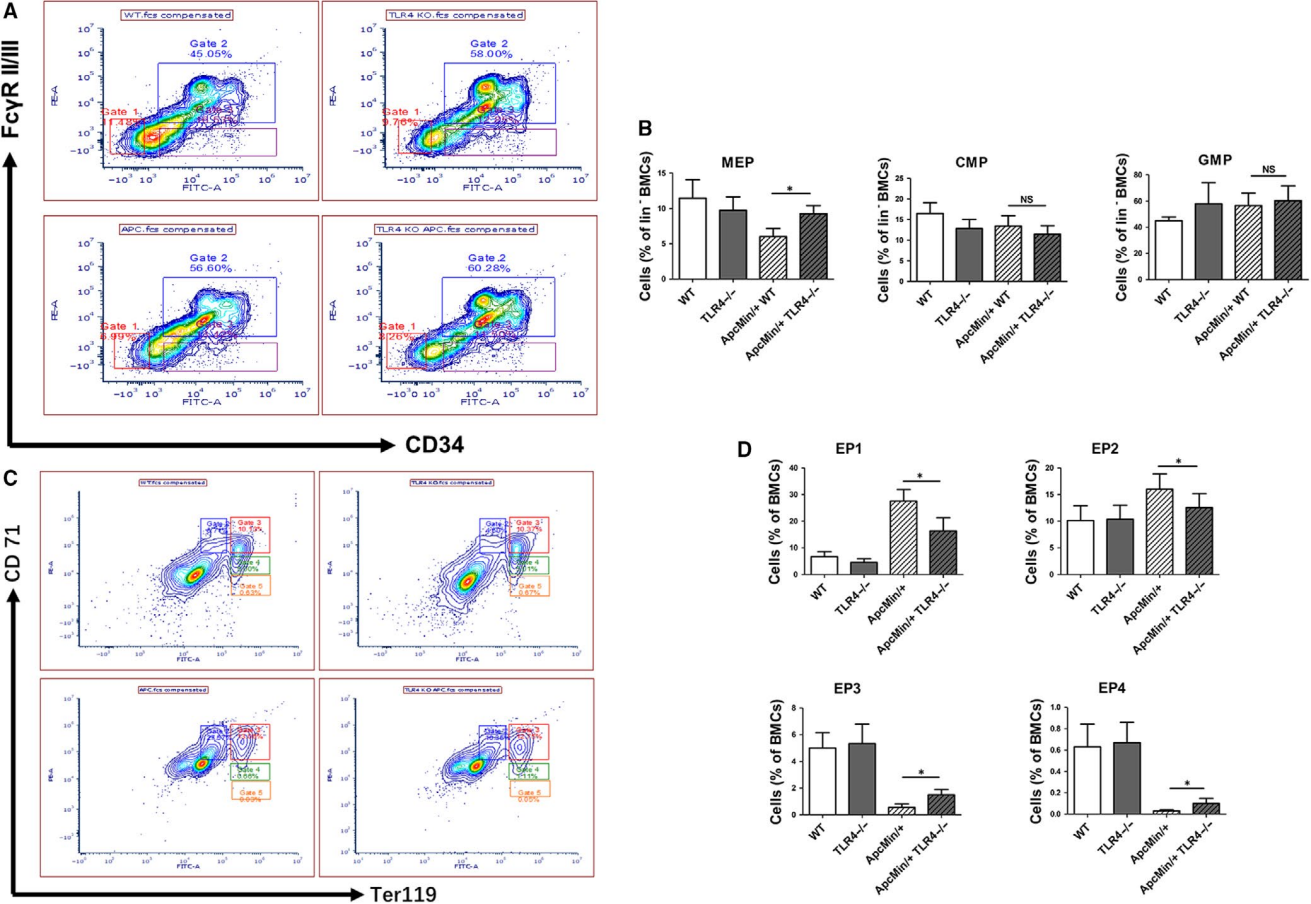
Amounts of bone marrow haematopoietic precursor cells at each stage of each group of mice were measured by flow cytometry. (A)Flow cytometry results of bone marrow precursor cells CMP(Lin^−^c‐Kit^+^FcγRII/III^lo^CD34^hi^), MEP (Lin^−^c‐Kit^+^FcγRII/III^lo^CD34^lo^) and GMP (Lin^−^c‐Kit^+^FcγRII/III^hi^CD34^hi^) of each group of mice. Gate1 is represented as MEP; Gate2 is represented as GMP; Gate3 is represented as CMP. (B) Statistical analysis of bone marrow precursor cells CMP, MEP and GMP in WT, TLR4^−/−^, Apc^Min/+^ and Apc^Min/+^ TLR4^−/−^ mice. (C) Flow cytometry results of bone marrow erythroid precursor cells EP1 (Ter119^−^CD71^hi^), EP2 (Ter119^hi^CD71^hi^), EP3 (Ter119^hi^CD71^med^) and EP4 (Ter119^hi^CD71^lo^) of each group of mice. Gate2 is represented as EP1; Gate3 is represented as EP2; Gate4 is represented as EP3; Gate5 is represented as EP4. (D) Statistical analysis of bone marrow erythroid precursor cells EP1, EP2, EP3 and Ep4 in WT, TLR4^−/−^, Apc^Min/+^, Apc^Min/+^ and TLR4^−/−^ mice. *P < .05; NS: No significant difference detected

**Figure 3:**
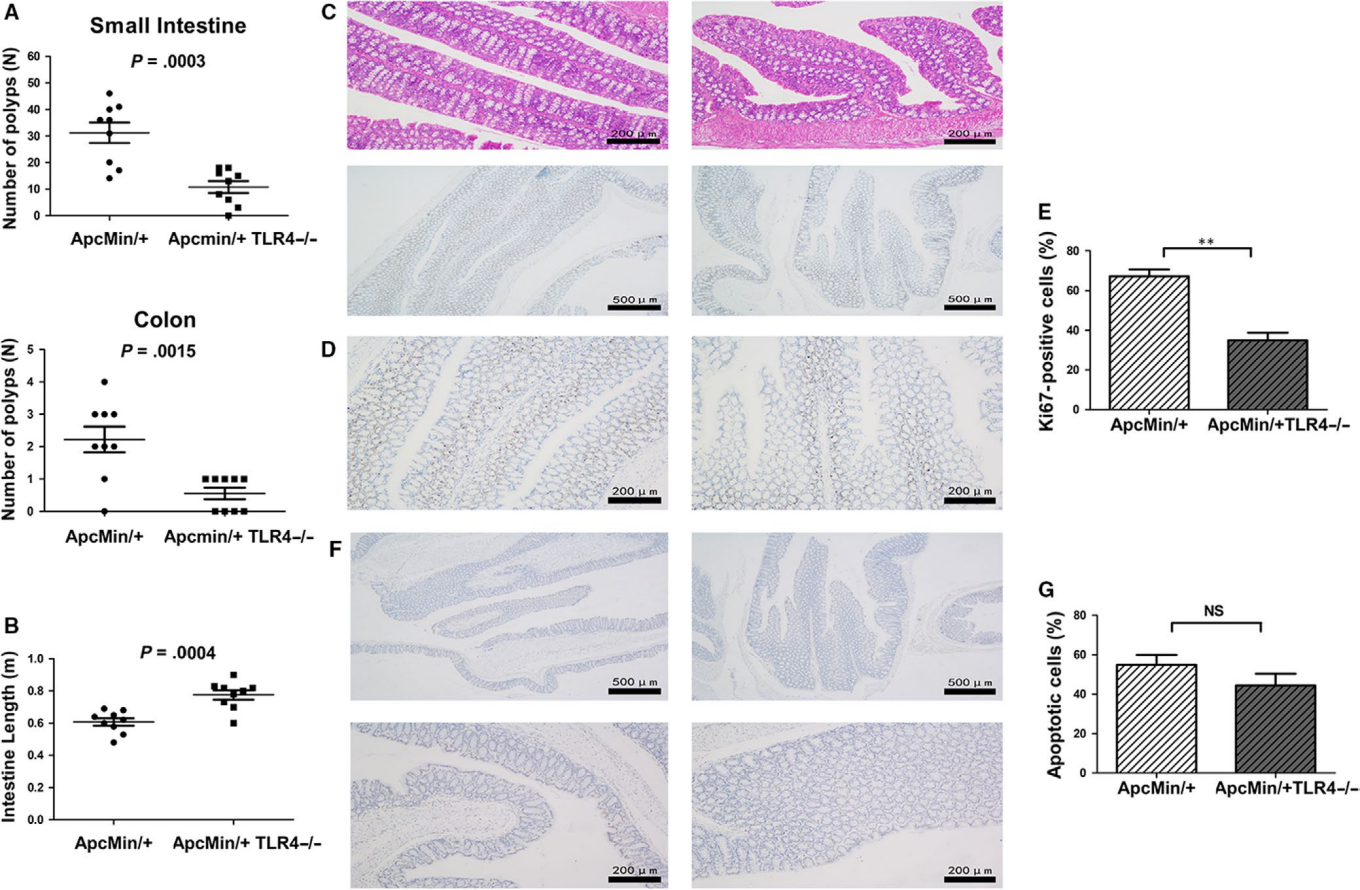
Numbers of intestinal polyps, intestinal length and histology studies in Apc^Min/+^WT and Apc^Min/+^ TLR4^−/−^ mice. (A) The number of visible polyps in the small intestine and colon was quantified via microscope in 20‐ to 22‐week‐old Apc^Min/+^WT and Apc^Min/+^ TLR4^−/−^ mice. (B) The intestinal lengths of 20‐ to 22‐week‐old Apc^Min/+^ WT and Apc^Min/+^ TLR4^−/−^ mice. (C) H&E staining photographs of intestines from 20‐ to 22‐week‐old Apc^Min/+^ WT and Apc^Min/+^ TLR4^−/−^ mice (×100 magnification). (D) Immunohistochemistry (Ki67) photographs of intestines from 20‐ to 22‐week‐old Apc^Min/+^ WT and Apc^Min/+^ TLR4^−/−^ mice (×40 and × 100 magnification). (E) The comparison of Ki67‐positive cells in intestines of Apc^Min/+^ WT and Apc^Min/+^ TLR4^−/−^ mice. (F) Terminal deoxynucleotidyl nick end labelling (TUNEL) stain of intestinal tissue from 20‐ to 22‐week‐old Apc^Min/+^ WT and Apc^Min/+^ TLR4^−/−^ mice (×40 and × 100 magnification). (G) The comparison of apoptotic cells in intestines of Apc^Min/+^ WT and Apc^Min/+^ TLR4^−/−^ mice. **P < .01; NS: No significant difference detected

Initially, to explore the role of TLR4 in spontaneous intestinal tumorigenesis, Apc^Min/+^ model mice sufficient and deficient in TLR4 were generated. It was found that Apc^Min/+^ TLR4^−/−^ mice had a longer survival time than Apc^Min/+^ mice (Figure 1A). Apc^Min/+^ mice died within 13.7‐25.5 weeks of age, and Apc^Min/+^ TLR4^−/−^ mice died within 16.8‐31.3 weeks of age. Some indices of peripheral blood in these mice were also explored, as they are markers of intestinal tumorigenesis. Apc^Min/+^ TLR4^−/−^ mice were significantly better than Apc^Min/+^ mice (Figure 1B‐F); TLR4 inhibited bone marrow haematopoietic stem cells, resulting in anaemia symptoms in Apc^Min/+^ mice. Bone marrow haematopoietic stem cells were detected in Apc^Min/+^ and Apc^Min/+^ TLR4^−/−^ mice by flow cytometry. Red blood cells (RBC), granulocytes and platelets are differentiated from common myeloid precursor cells (CMPs), and CMPs continue to differentiate into megakaryocyte‐erythroid progenitors (MEP) and granulocyte‐macrophage progenitors (GMP). Therefore, the number changes of CMP, MEP and GMP in the bone marrow cells of these mice were examined. The results revealed that the mean proportion of MEP in bone marrow cells (BMCs) was 6.35% in Apc^Min/+^ WT mice, which was lower than 9.2% in Apc^Min/+^TLR4^−/−^ mice (*P* < .05) (Figure 2A,B). However, the mean proportion of CMP or GMP in BMCs was no significant statistic difference between Apc^Min/+^ WT and Apc^Min/+^TLR4^−/−^ mice (Figure 2A,B). To further investigate the cause of elevated Apc^Min/+^ TLR4^−/−^ peripheral red blood cells, the numbers of erythroid precursor cells of bone marrow in each group were examined. The development of RBC is mainly derived from four stages: proerythroblast (EP1), prorubricyte (EP2), polychromatic erythroblast (EP3), and metarubricyte (EP4). The flow cytometry results showed that Apc^Min/+^ TLR4^−/−^ erythrocyte precursors were higher than those of Apc^Min/+^ WT in the EP3 and EP4 stages (Figure 2C,D). The numbers of visible polyps in the small intestines and colons of the Apc^Min/+^ and Apc^Min/+^ TLR4^−/−^ mice were then observed. The number of macroadenomas in the Apc^Min/+^ TLR4^−/−^ mice was reduced compared with that in the Apc^Min/+^ controls (Figure 3A). Additionally, the intestine lengths of the Apc^Min/+^ TLR4^−/−^ mice were generally longer than those of the Apc^Min/+^ WT mice (Figure 3B). Microscopic evaluations were also conducted to explore the inhibition of the formation of intestinal adenomas in Apc^Min/+^ mice deficient in TLR4. The Apc^Min/+^ TLR4^−/−^ group displayed fewer tumours than the Apc^Min/+^ WT group (Figure 3C). The numbers of Ki67‐positive cells were much higher in gut samples from the Apc^Min/+^ WT mice than in samples from the Apc^Min/+^ TLR4^−/−^ mice (Figure 3D,E). However, there was no difference in the numbers of apoptosis cells in the colon samples of Apc^Min/+^ WT and Apc^Min/+^ TLR4^−/−^ mice (Figure 3F,G).

### 3.2 Expression patterns and KEGG pathway enrichment analysis of differential genes in gut tumours of Apc^Min/+^ WT controls and Apc^Min/+^ TLR4^−/−^ mice

**Figure 4:**
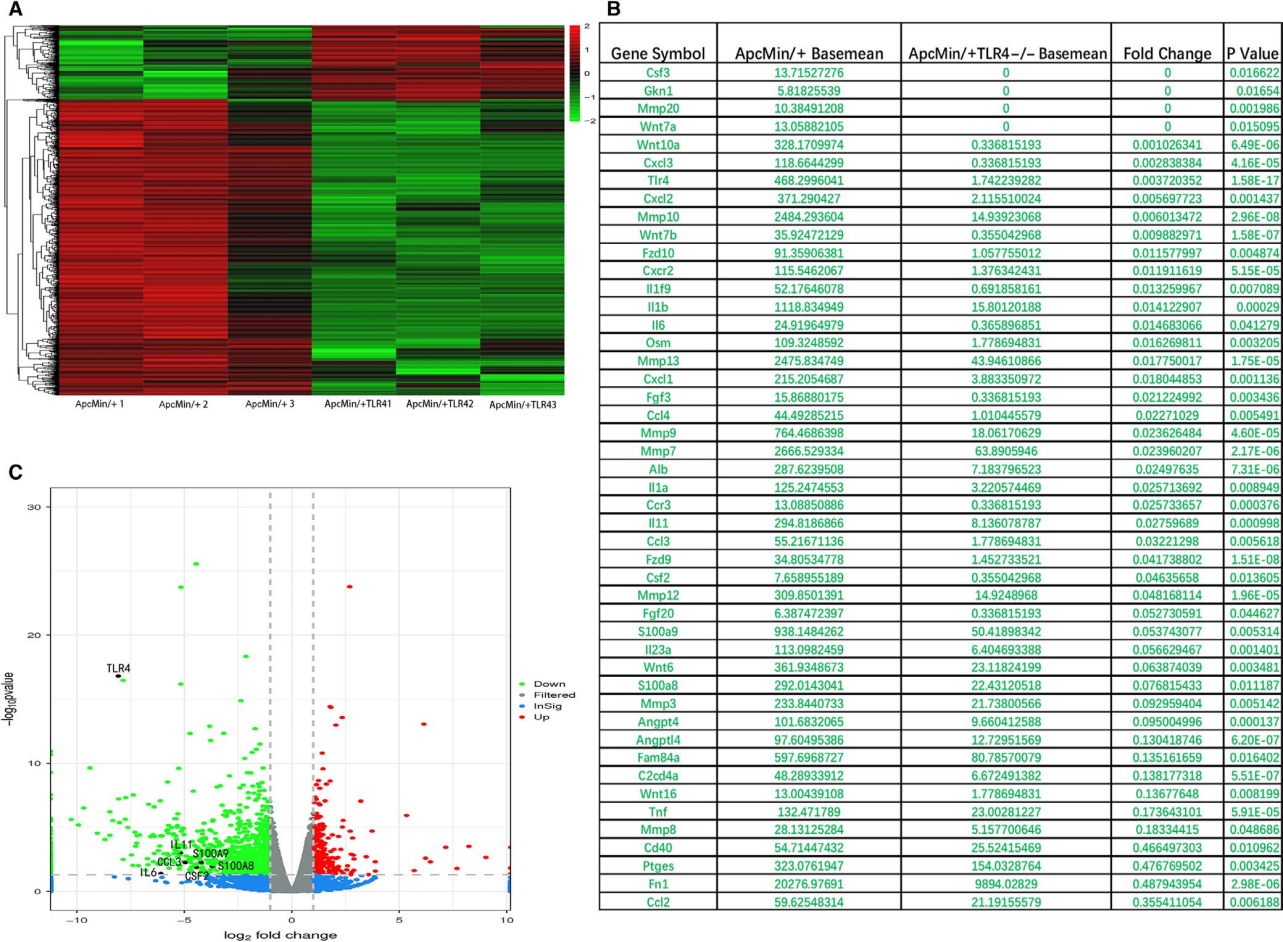
Differential genes in intestinal tumours were analysed by high‐throughput RNA‐Seq. (A) Cluster analysis chart. Red indicates that genes were up‐regulated, and green indicates that genes were down‐regulated. (B) The obvious down‐regulated differential genes of concern in Apc^Min/+^ TLR4^−/−^ model mice were selected. (C) Volcano plot that displays the dysregulated genes in intestinal tumours of Apc^Min/+^ WT and Apc^Min/+^ TLR4^−/−^ mice. The vertical grey lines correspond to twofold up‐ and down‐regulation, and the horizontal grey line represents a *P*‐value of 0.05. The red points indicate up‐regulated genes in tumours of Apc^Min/+^ TLR4^−/−^ mice, and the green points indicate down‐regulated genes in tumours of Apc^Min/+^ TLR4^−/−^ mice

To determine how TLR4^−/−^inhibited intestinal tumorigenesis, the global gene expression profiles in the gut tumours of the Apc^Min/+^ WT controls and Apc^Min/+^ TLR4^−/−^ mice were compared. A cut‐off value of a log2 fold change greater than 1 and a *P*‐value less than 0.05 were used to extract the differentially expressed genes. A total of 1248 differential genes were detected by RNA‐Seq in the 3 pairs of colon samples from the Apc^Min/+^ WT and Apc^Min/+^ TLR4^−/−^ mice. A cluster analysis of differentially expressed genes in three replicated Apc^Min/+^ WT and Apc^Min/+^ TLR4^−/−^ mice intestinal samples was performed. The results suggest that the gene expression patterns between them were distinguishable (Figure 4A). We selected 47 down‐regulated differential genes with fold change greater than 2 and *P*‐value less than 0.05 in the Apc^Min/+^ TLR4^−/−^ model mice (Figure 4B). The selected genes were more commonly used in our experimental system and had a certain role in promoting tumour proliferation. Volcano plots were created to visualize the significant differential up‐regulation and down‐regulation genes in the gut tumours of Apc^Min/+^ TLR4^−/−^ mice compared with Apc^Min/+^ WT mice (Figure 4C). A differential gene KEGG pathway enrichment analysis was also conducted. Analysis of the KEGG database revealed differences between the Apc^Min/+^ WT and Apc^Min/+^ TLR4^−/−^ mice in 255 pathways. The top 20 total pathways with a number of differential genes greater than 2 (Figure S1A) were screened out, and it was found that differential genes were much more expressed in two pathways: cytokine‐cytokine receptor interaction and pathways in cancer (Figure S1B) (*P* < .001). The molecule in the dark green and red box, respectively, indicates that the expressions of this molecule in the intestinal tumours in Apc^Min/+^ TLR4^−/−^ mice were significantly lower and higher than in the intestinal tumours in Apc^Min/+^ WT mice. The green box indicates that the expression of this molecule in the Apc^Min/+^ WT and Apc^Min/+^ TLR4^−/−^ mice was not significantly different. The expressions of important regulatory factors CXCL1, CXCL2, CCL2, CSF2, CSF3, IL6 and IL11 in the cytokine‐cytokine receptor interaction pathway were significantly lower in the Apc^Min/+^ TLR4^−/−^ mice gut tissue than in the Apc^Min/+^ WT control gut tissue. In the pathways in cancer, the expression of Wnt signalling was significantly decreased in the Apc^Min/+^ TLR4^−/−^ mice gut tissue compared with the Apc^Min/+^ WT control. It has been reported that Wnt signalling promotes the development of colon cancer.^28^ Based on the above KEGG data, TLR4^−/−^ might slow down intestinal tumorigenesis by attenuating cytokine‐cytokine receptor interaction and pathways in cancer.

### 3.3 **The key down‐regulated genes in gut tumours of Apc^Min/+^TLR4** ^−/−^ **model mice were validated**

**Figure 5:**
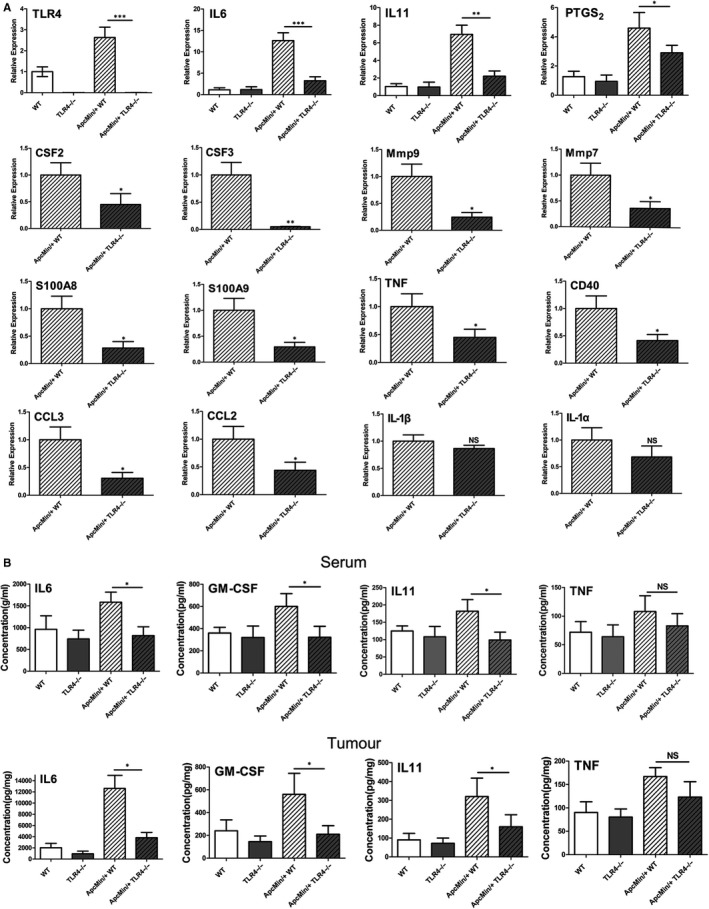
Validation assays of down‐regulated differential genes that were of concern in intestinal tumours of Apc^Min/+^ TLR4^−/−^ mice. (A) RT‐qPCR validation of relative expressions of 16 differential genes in tumours of each group of mice. (B) ELISA validation of IL‐6, TNF, IL‐11 and GM‐CSF in the serum and tumours of each group of mice. *P < .05, **P < .01, ***P < .001; NS: No significant difference detected

RT‐qPCR was then performed for the 16 concerned obviously down‐regulated genes, which were selected from dysregulated differential genes. These 16 genes were significantly down‐regulated in Apc^Min/+^ TLR4^−/−^ intestinal tumours as determined by RNA‐Seq analysis and were commonly used in the experimental system of the study. The expressions of TLR4, IL‐6, IL‐11 and PTGS2 in size‐matched polyps were compared between the WT, TLR4^−/−^, Apc^Min/+^ WT and Apc^Min/+^ TLR4^−/−^ mice groups, and the expressions of the other genes were compared between the Apc^Min/+^ WT and Apc^Min/+^ TLR4^−/−^ groups. The results were consistent with the RNA‐Seq results; compared with the Apc^Min/+^ controls, the selected genes were all down‐regulated in polyps of Apc^Min/+^ TLR4^−/−^ mice. TLR4, IL‐6, IL‐11 and CSF3 expressions in the Apc^Min/+^ TLR4^−/−^ groups were down‐regulated significantly (Figure 5A). ELISA was performed to measure the IL‐6, TNF, IL‐11 and GM‐CSF expression levels in the serum or in the intestinal polyps of the WT, TLR4^−/−^, Apc^Min/+^ WT and Apc^Min/+^ TLR4^−/−^ mice. The results suggest that, compared with Apc^Min/+^ WT mice, IL6, GM‐CSF and IL11 were down‐regulated in Apc^Min/+^ TLR4^−/−^ mice, and the genes generated by intestinal polyps were more significantly down‐regulated (Figure 5B). Additionally, 13 factors in the serum were compared between Apc^Min/+^ and Apc^Min/+^ TLR4^−/−^ mice by flow multifactor detection assay. Compared with the Apc^Min/+^ WT mice, these 13 cytokines decreased to some degree in the Apc^Min/+^ TLR4^−/−^ mice, and IL‐6 and GM‐CSF down‐regulation was also more obvious (Figure S2A,B).

### 3.4 IL‐6 and GM‐CSF were mediated by TLR4 in WT or Apc^Min/+^ mice in protein levels

**Figure 6:**
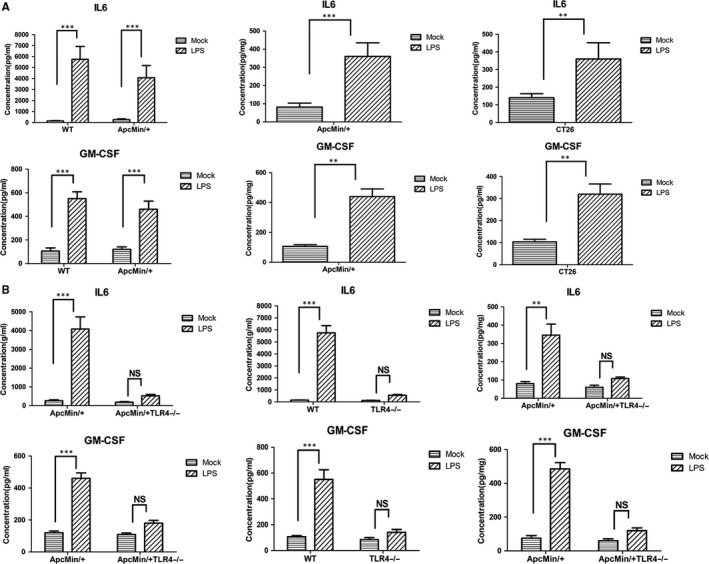
IL‐6 and GM‐CSF were mediated by TLR4 in WT or Apc^Min/+^ mice. (A) The left panels are, respectively, the expression levels of IL6 and GM‐CSF in the serum of WT with or without LPS treatment (1 mg/kg bodyweight), and Apc^Min/+^ mice with or without LPS treatment (1 mg/kg bodyweight). The middle panels are the expression levels of IL6 and GM‐CSF in the colons of Apc^Min/+^ mice with or without LPS treatment (1 mg/kg bodyweight). The right panels are the expression levels of IL6 and GM‐CSF in CT‐26 cells with or without LPS treatment (1 ng/mL). (B) The left panels are, respectively, the expression levels of IL6 and GM‐CSF in the serum of Apc^Min/+^ mice with or without LPS treatment (1 mg/kg bodyweight), and Apc^Min/+^ TLR4^−/−^ mice with or without LPS treatment (1 mg/kg bodyweight). The middle panels are, respectively, the expression levels of IL6 and GM‐CSF in the serum of WT mice with or without LPS treatment (1 mg/kg bodyweight), and TLR4^−/−^ mice with or without LPS treatment (1 mg/kg bodyweight). The right panels are, respectively, the expression levels of IL6 and GM‐CSF in the colons of Apc^Min/+^ mice with or without LPS treatment (1 mg/kg bodyweight), and Apc^Min/+^ TLR4^−/−^ mice with or without LPS treatment (1 mg/kg bodyweight). *P < .05, **P < .01, ***P < .001; NS: No significant difference detected. All data were detected by ELISA assay

To explore the expression levels of IL6 and GM‐CSF regulated by TLR4 signalling, three levels of experimental studies were conducted: in the serum and colons of the mice, and in the colon cancer cells. LPS (TLR4 agonist) significantly activated the expression levels of IL6 and GM‐CSF in the serum of WT mice. The expression levels of IL6 and GM‐CSF in the serum and colons of Apc^Min/+^ mice were also increased by LPS activation. Simultaneously, the expression levels of IL6 and GM‐CSF in the CT26 cells were also significantly increased by LPS stimulation (Figure 6A). Compared with WT mice, the expression levels of IL6 and GM‐CSF in the serum of TLR4^−/−^ mice presented no significant change with LPS treatment. Also, compared with Apc^Min/+^WT mice, the expression levels of IL6 and GM‐CSF in the serum or colons of Apc^Min/+^TLR4^−/−^ mice presented no significant change with LPS treatment (Figure 6B). These data suggest that the expressions of IL6 and GM‐CSF were regulated by TLR4 signalling in protein levels.

### 3.5 Functional study of down‐regulation factors such as IL6 and GM‐CSF in intestinal cancer cell lines

**Figure 7:**
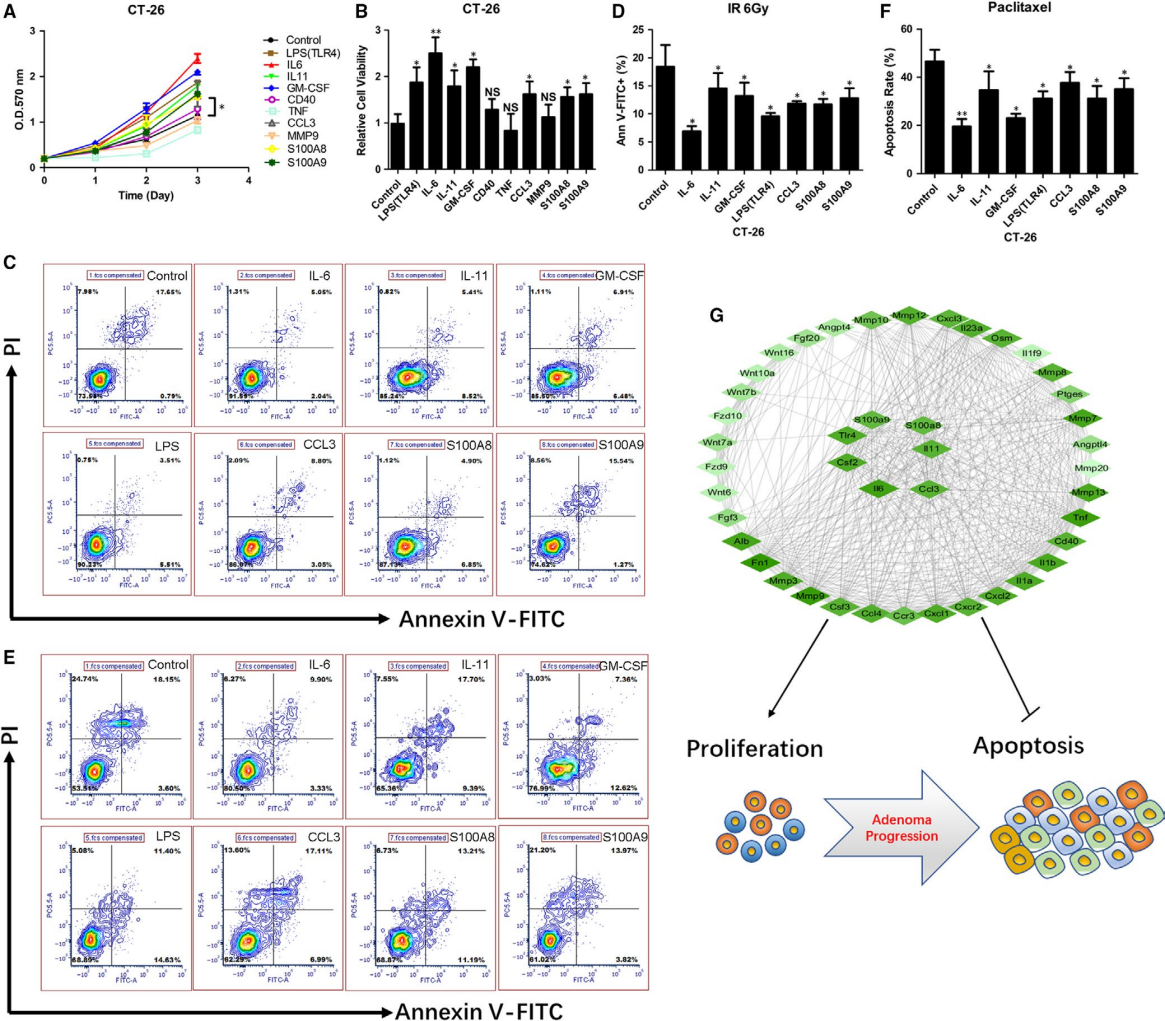
Functional study of down‐regulation factors in mouse intestinal cancer CT‐26 cell line. (A) CT‐26 cells were treated with 100 ng/mL LPS, IL6, IL11, GM‐CSF, CD40, TNF, CCL3, MMP9 and S100A8/9, and the cell viability was detected at days 0, 1, 2 and 3 by MMT assay. (B) Statistical analysis of cell viability of cytokines‐treated CT‐26 cells. (C) CT‐26 cells were treated with 100 ng/mL LPS, IL6, IL11, GM‐CSF, CCL3 and S100A8/9 for 24 h, then exposed to 6 Gy radiation. After 24 h, the apoptosis rates of cytokines‐treated CT‐26 cells were detected with Annexin V/PI double staining by flow cytometry. (D) Statistical analysis of Ann V‐FITC positive of cytokines‐treated CT‐26 cells with 6 Gy radiation. (E) CT‐26 cells were treated with 100 ng/mL LPS, IL6, IL11, GM‐CSF, CCL3 and S100A8/9 for 24 h, then exposed to paclitaxel. After 24 h, the apoptosis rates of cytokines‐treated CT‐26 cells were detected with Annexin V/PI double staining by flow cytometry. (F) Statistical analysis of apoptosis rates of cytokines‐treated CT‐26 cells with paclitaxel. (G) Network interaction map of TLR4 and its core downstream factors promoting spontaneous intestinal tumorigenesis.*P < .05, **P < .01, NS: No significant difference detected

CT‐26 cells belong to the mouse intestinal cancer cell line. CT‐26 cells were treated with LPS (TLR4 agonist), IL‐6, IL‐11, GM‐CSF, CD40, TNF, CCL3, MMP9, S100A8 and S100A9, and cell viability was detected after 24 h using MTT assay. The results showed that, except for TNF, MMP9 and CD40, the other cytokines effectively enhanced CT‐26 cell viability, especially cytokines TLR4, IL‐6 and GM‐CSF (Figure 7A,B). Next, the apoptosis rates of the cytokines‐treated CT‐26 cells were examined after the administration of radiation and the chemotherapeutic drug paclitaxel by Annexin V/PI double staining. It was found that, compared with the control, the apoptosis rates of cytokines‐treated CT‐26 cells decreased after radiation and chemotherapy (Figure 7C‐F). Similar experiments were also conducted on the human intestinal cancer cell line SW1116 cell. It was found that IL6, GM‐CSF, IL11 and LPS effectively enhanced SW1116 cell viability (Figure S3A,B), and, compared with the control, the apoptosis rates of IL6, GM‐CSF, IL11 and LPS‐treated SW1116 cells decreased after radiation and chemotherapy (Figure S3C,D). These data show that the inhibition of the activity of cytokines such as IL‐6 and GM‐CSF could effectively improve the sensitivity of radiotherapy and chemotherapy to colon cancer cells. Based on these results, a network map of TLR4 and its regulatory genes that promoted mouse gut tissue proliferation and apoptosis in intestinal tumour formation and development was constructed (Figure 7G).

## 4 DISCUSSION

Vogelstein et al^29^ held that patients with germline mutations of Apc did not necessarily develop CRC, along with additional risk factors, such as inflammation induction leading to tumour development. TLR4 pathway‐mediated immune response is an important mechanism that aggravates intestinal injury in mouse ulcerative colitis models.^30^ In the present study, Apc^Min/+^ intestinal adenoma mice were generated on TLR4‐sufficient and TLR4‐deficient backgrounds to investigate the carcinogenic effect of TLR4 by comparing mice survival, peripheral blood cells, bone marrow haematopoietic precursor cells and numbers of polyps in the guts of Apc^Min/+^ WT and Apc^Min/+^ TLR4 mice. The results revealed that TLR4 had a critical role in promoting spontaneous intestinal tumorigenesis. To study the downstream genes involved in the promotion of intestinal tumorigenesis by TLR4 signalling, high‐throughput RNA sequencing was utilized to screen for dysregulated genes in gut tumours between Apc^Min/+^ WT and Apc^Min/+^ TLR4 mice. After a series of validation experiments for the concerned genes, it was found that IL6, GM‐CSF (CSF2), IL11, CCL3, S100A8 and S100A9 were significantly decreased in the gut tumours of Apc^Min/+^ TLR4^−/−^ mice compared with Apc^Min/+^ WT mice. Combined with the KEGG enrichment data, it can be determined that TLR4 might promote intestinal tumorigenesis by activating cytokine‐cytokine receptor interaction and pathways in cancer signalling pathways. In the functional study of core down‐regulation factors, it was found that IL6, GM‐CSF, IL11, CCL3 and S100A8/9 increased the viability and decreased the apoptosis rate of colon cancer cells with irradiation and chemical treatment. The effects of IL6 and GM‐CSF were the most obvious.

The tumour‐promoting effect of inflammation is now widely recognized and better understood.^31^ The NF‐κB‐IL6‐Stat3 cascade is an important inflammatory regulator of the proliferation of tumour‐initiating intestinal epithelial cells.^32^ Baltgalvis et al^33^ reported that IL6 was essential for the development of cachexia in Apc^Min/+^ WT mice and was associated with a greater tumour burden. Apc^Min/+^ WT mice with severe cachectic symptoms and intestinal polyp burdens had high expression levels of IL6 and overexpression of IL6, which induced skeletal muscle wasting and polyp formation. In the present experiment system, the intestinal tumorigenesis was alleviated in Apc^Min/+^ TLR4^−/−^ mice, and the expression of IL6 was decreased significantly in the serum or gut tumours of Apc^Min/+^ TLR4^−/−^ mice compared with Apc^Min/+^ WT mice. Simultaneously, LPS (TLR4 agonist) significantly up‐regulated the expression levels of IL6 in the serum or colons of Apc^Min/+^ WT mice and in the CT26 cells. However, the expression levels of IL6 activated by LPS presented no significant changes in WT or Apc^Min/+^ TLR4^−/−^ mice. These data suggest that TLR4 was the upstream molecule of IL6 that promoted tumour development, and it has been reported that TLR4 and NF‐κB signalling are required to control the regulation of the IL‐6 mRNA stabilizing molecule Arid5a.^34^

GM‐CSF was initially classified as a haematopoietic growth factor. In the inflammatory process, GM‐CSF serves as a communication conduit between tissue‐invading lymphocytes and myeloid cells, and GM‐CSF‐activated phagocytes are well equipped to cause tissue damage.^35^ Chen et al^36^ reported that GM‐CSF‐treated cancer cells exhibited an enhanced ability of motility both in vitro and in vivo, and that chronic exposure of intestinal cancer cells to GM‐CSF led to the occurrence of intestinal epithelium to mesenchymal transition (EMT). Ripk3 signalling promotes intestinal tumours by up‐regulating cytokines IL23 and IL1β, which are required for expanding IL‐17‐producing T cells through I‐MDSCs, a distinct MDSC subset that is dependent on GM‐CSF.^37^ Ripk3 activity is mediated by TLR4.^38^ We ever used CD11b/Gr_1_ double staining to detect the expression of MDSC in spleen and bone marrow of WT, Apc^Min/+^, Apc^Min/+^TLR4^−/−^ mice by flow cytometry. The results suggested that the expression level of MDSC in spleen and bone marrow of Apc^Min/+^ mice was higher than that in WT or Apc^Min/+^TLR4^−/−^ mice (Figure S4A,B). These data proved MDSC was dependent on TLR4. The current experiment system suggests that LPS activated the expression level of GM‐CSF in the serum or colons of Apc^Min/+^ mice and in the CT26 cells. However, the expression levels of GM‐CSF activated by LPS presented no significant changes in WT or Apc^Min/+^ TLR4^−/−^ mice. Combined with the RNA‐Seq data and validation results, it can be determined that the expression levels of GM‐CSF were down‐regulated significantly in the gut tissues of Apc^Min/+^TLR4^−/−^ mice. It is hypothesized that the promotion of intestinal tumours by Ripk3/I‐MDSCs was mediated by TLR4/GM‐CSF signalling.

Chemotherapy and radiation therapy are important components of CRC treatment systems. Preoperative neoadjuvant chemoradiotherapy can convert unresectable tumours into resectable tumours. Postoperative adjuvant chemoradiotherapy can reduce the risk of local recurrence and distant metastasis.^39^ Through apoptosis experiments of the mouse intestinal cancer cell CT‐26 and the human intestinal cancer cell SW1116, it was found that IL‐6, IL‐11 and GM‐CSF, etc all inhibited the cell line apoptosis under radiation and chemical treatments. This indicates that the inhibition of the activity of cytokines such as IL6 and GM‐CSF could effectively improve the sensitivity of radiotherapy and chemotherapy to colon cancer cells.

## CONFLICTS OF INTEREST

The authors have no competing interests to declare.

## AUTHOR CONTRIBUTIONS

Hao Wang and Cong Liu designed the overall study with contributions from Yun‐Jie Shi. Yun‐Jie Shi and Quan‐Quan Zhao designed and carried out experiments, collected and analysed data. Xiao‐Shuang Liu and Su‐He Dong designed the vector for the repair experiment. Cong Liu constructed the repair vector. Yun‐Jie Shi, Ji‐Fu E and Xu Li carried out the repair experiment. Yun‐Jie Shi edited the paper. The paper was discussed and revised by Hao Wang and Cong Liu.

## Data Availability

This is an open access article under the terms of the Creative Commons Attribution License, which permits use, distribution and reproduction in any medium, provided the original work is properly cited.
