# Supplementary figures and images for: Toll‐like receptor 4 regulates spontaneous intestinal tumorigenesis by up‐regulating IL‐6 and GM‐CSF
Source: J Cell Mol Med. 2019 Oct 25;24(1):385–97. doi: 10.1111/jcmm.14742 (PMC6933338; doi:10.1111/jcmm.14742)

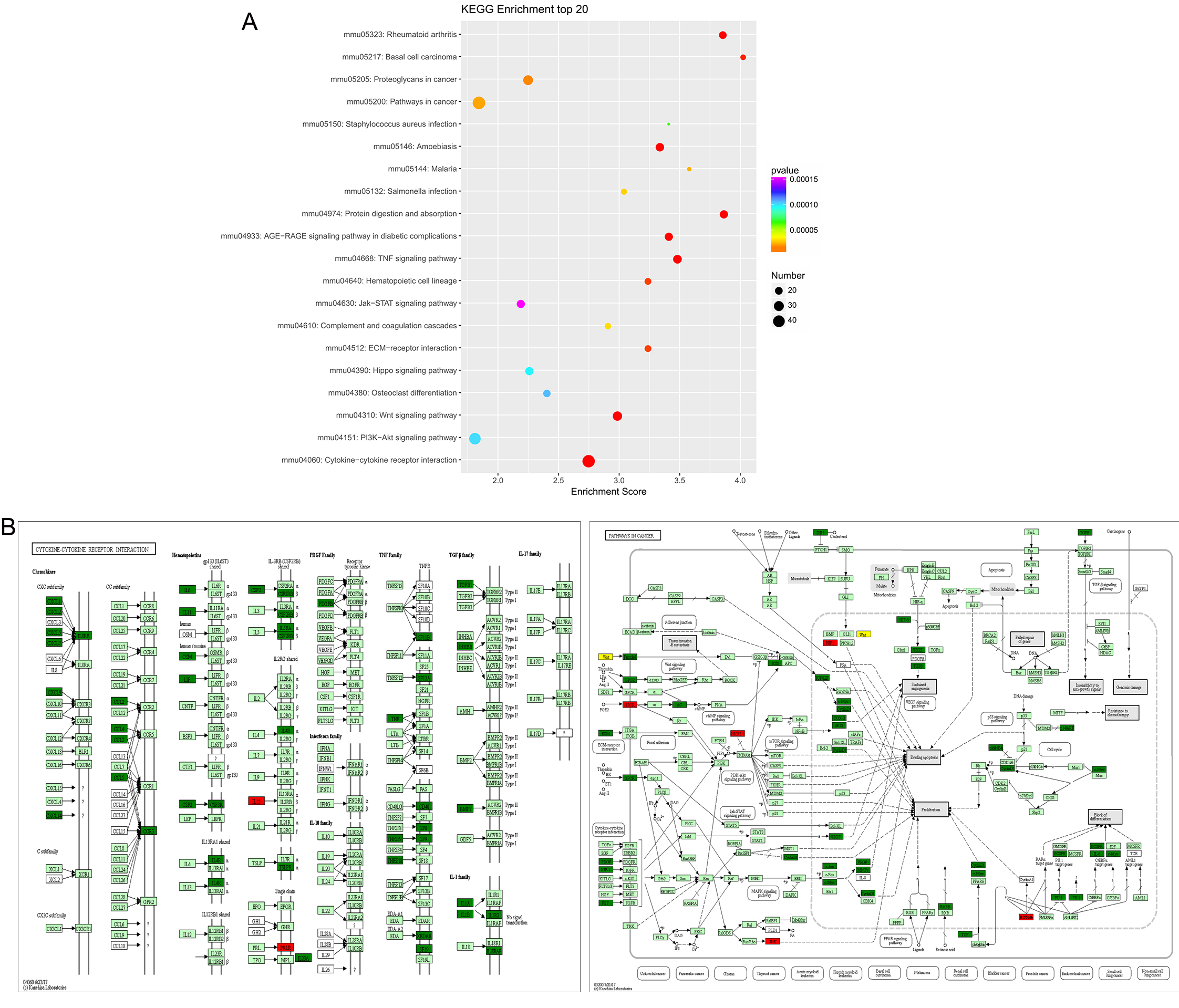

Supplement: Supplementary file 1 [file JCMM-24-385-s001.tif]

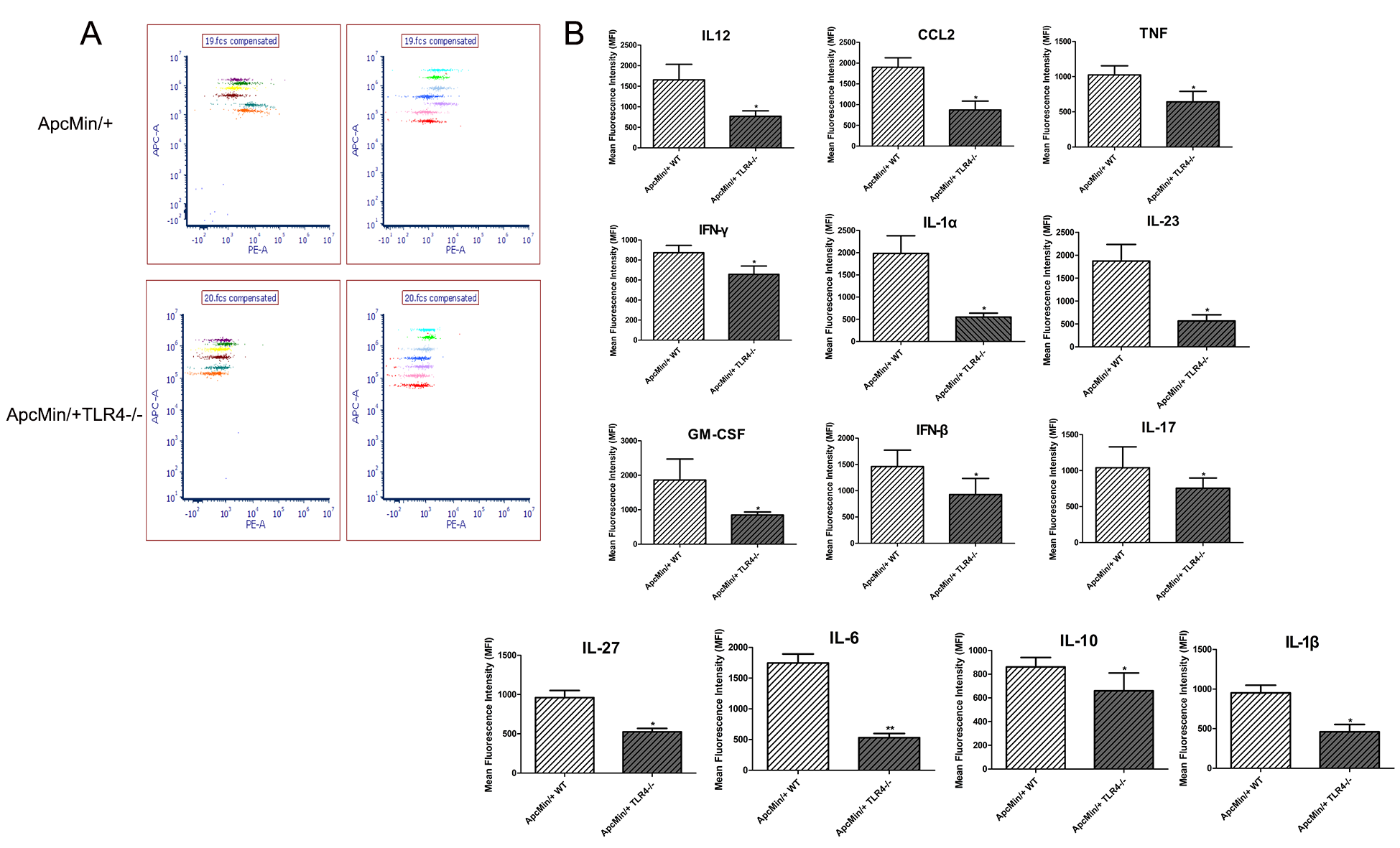

Supplement: Supplementary file 2 [file JCMM-24-385-s002.tif]

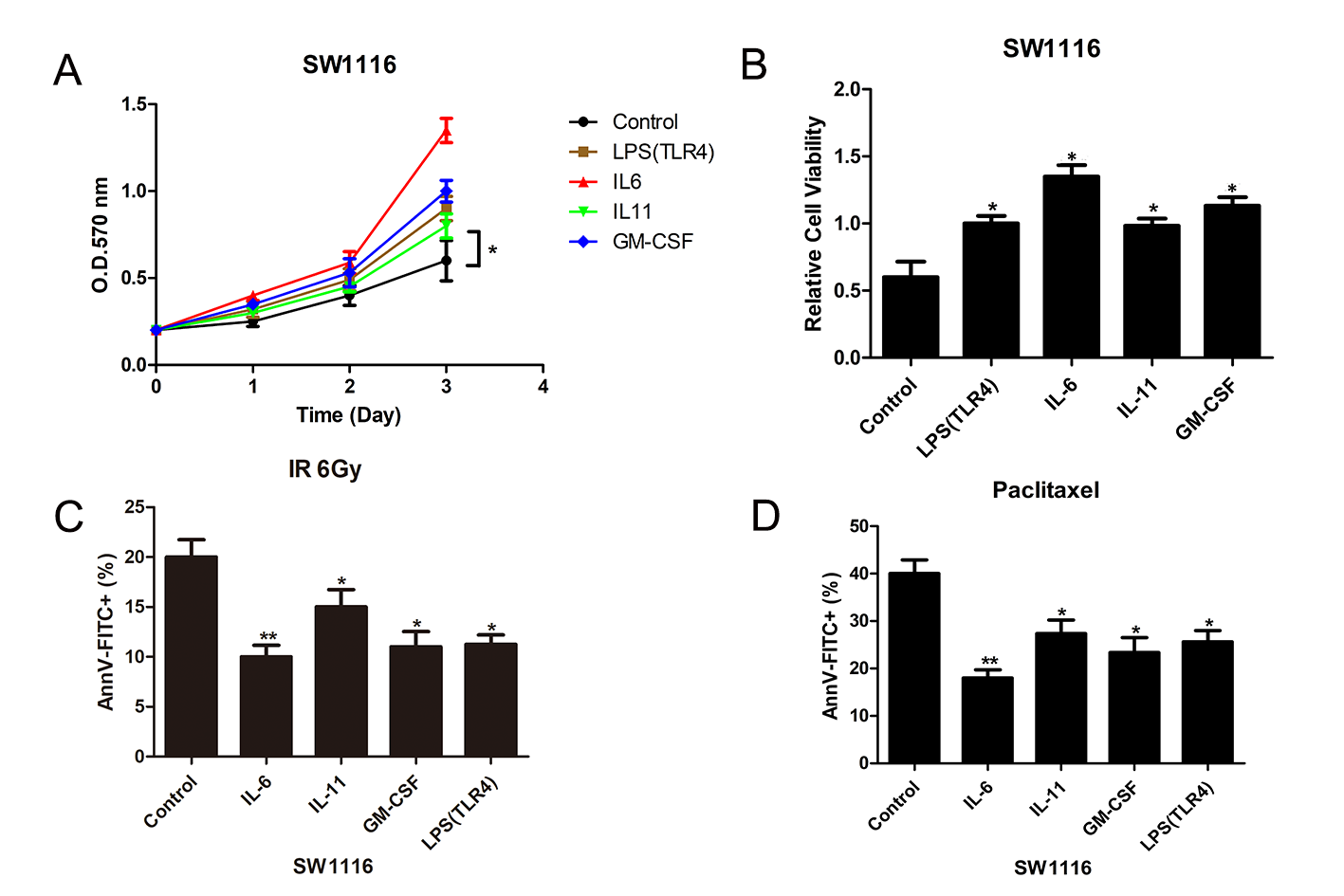

Supplement: Supplementary file 3 [file JCMM-24-385-s003.tif]

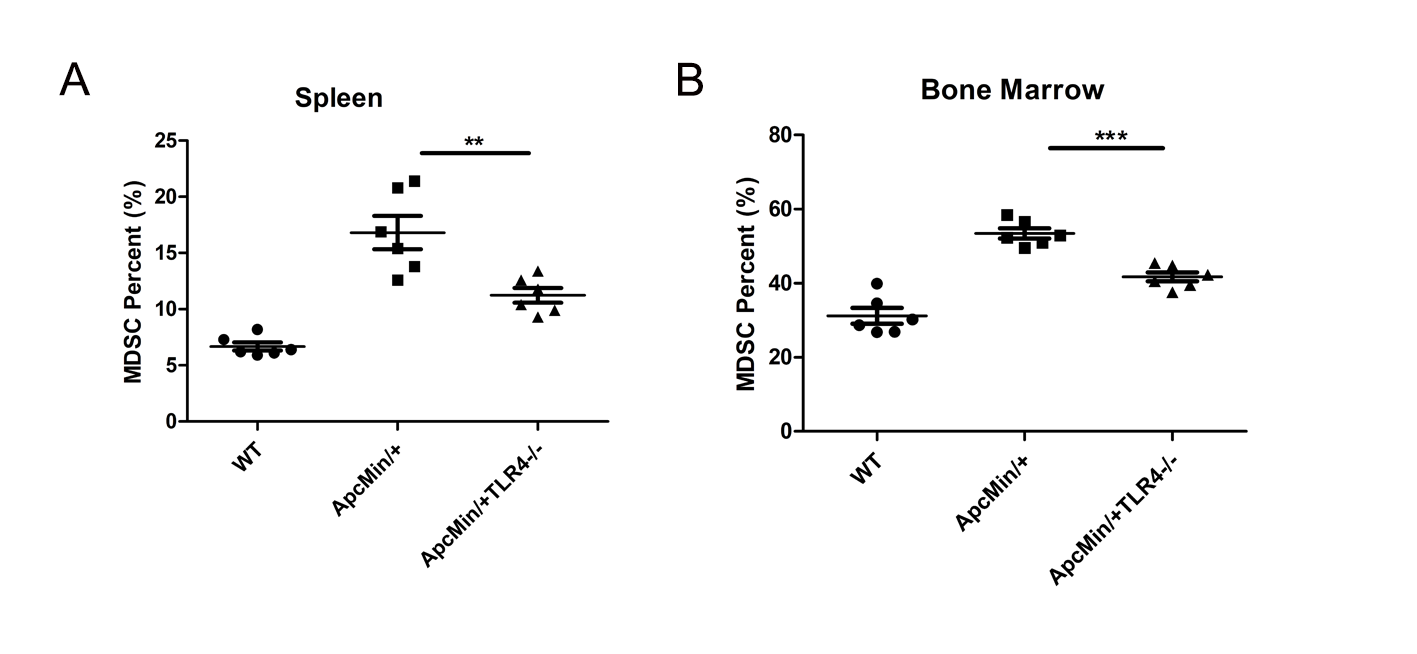

Supplement: Supplementary file 4 [file JCMM-24-385-s004.tif]
